# Satellite tracking reveals novel migratory patterns and the importance of seamounts for endangered South Pacific humpback whales

**DOI:** 10.1098/rsos.150489

**Published:** 2015-11-25

**Authors:** Claire Garrigue, Phillip J. Clapham, Ygor Geyer, Amy S. Kennedy, Alexandre N. Zerbini

**Affiliations:** 1 Opération Cétacés, Nouméa, New Caledonia; 2 Institut de Recherche pour le Développement UMR ENTROPIE, IRD, Perpignan, France; 3 National Marine Mammal Laboratory, Alaska Fisheries Science Center, Seattle, WA, USA; 4 Instituto Aqualie, Projeto Monitoramento de Baleias por Satélite, Rio de Janeiro, Brazil; 5 Cascadia Research Collective, Olympia, WA, USA

**Keywords:** whale, distribution, cryptic habitat, satellite tracking, Oceania, breeding ground

## Abstract

The humpback whale population of New Caledonia appears to display a novel migratory pattern characterized by multiple directions, long migratory paths and frequent pauses over seamounts and other shallow geographical features. Using satellite-monitored radio tags, we tracked 34 whales for between 5 and 110 days, travelling between 270 and 8540 km on their southward migration from a breeding ground in southern New Caledonia. Mean migration speed was 3.53±2.22 km h^−1^, while movements within the breeding ground averaged 2.01±1.63 km h^−1^. The tag data demonstrate that seamounts play an important role as offshore habitats for this species. Whales displayed an intensive use of oceanic seamounts both in the breeding season and on migration. Seamounts probably serve multiple and important roles as breeding locations, resting areas, navigational landmarks or even supplemental feeding grounds for this species, which can be viewed as a transient component of the seamount communities. Satellite telemetry suggests that seamounts represent an overlooked cryptic habitat for the species. The frequent use by humpback whales of such remote locations has important implications for conservation and management.

## 1. Introduction

Understanding the patterns of movement of free-ranging animals is crucial to assessing their habitat use and is therefore a prerequisite in the development of conservation management strategies. Because of the tendency of humpback whales (*Megaptera novaeangliae*) to come close to the coastline, at-sea studies of this species in the post-whaling era have focused largely on coastal populations. As such, there is a lack of information [1] on how, or whether, this species uses offshore areas such as seamounts [2]. Consequently, research is needed to assess the behaviour and habitat use of whales in such areas, and the relevance of offshore habitats for particular life-history stages of the species. It is also important to identify potential anthropogenic effects or impacts in these locations. Information of this nature is important to identify high-priority habitats for the development of conservation measures, such as designation as offshore Marine Protected Areas [3–5].

Seamounts are ubiquitous undersea mountains rising at least 100 m from the ocean seafloor [6,7]. These underwater features have recently been recognized as one of the largest biomes in the world [8] and a global assessment of knowledge regarding seamount ecosystems was recently conducted [9]. Seamounts support a large number of organisms and strongly influence the distribution of a wide range of species at different stages of their life cycle [10–14]. They play an important role for large predators [15–17] and air-breathing visitors [18,19], and a significant association between marine mammal abundance and seamount-rich locations has been established [20]. Seamounts have been seen as an aggregation point for highly migratory pelagic species [21–23], and recent studies combining environmental data with visual or acoustic surveys have shown that they are important foraging habitats for some cetaceans [11,24–26]. In spite of the difficulties of determining marine mammal habitat usage on larger geographical and temporal scales in oceanic areas, studies are needed to assess the reliance of individual species on seamounts and to explore the range of roles that this ecosystem type could represent [27].

In the past 20 years, satellite telemetry of humpback whales has provided a greater understanding of dispersal movements in wintering and summering areas in both hemispheres [28,29]. Some of these studies have provided data on the coastal and oceanic migratory paths followed by whales [2,30–34], but none of the studies demonstrated the regular use of seamounts. More recently, satellite telemetry revealed the existence of an unknown offshore habitat [35] for endangered southwestern Pacific Ocean humpback whales off New Caledonia [36].

Here we report on movements of satellite-monitored humpback whales in their breeding grounds off New Caledonia and examine the southbound migration from low-latitude coastal habitats in relation to topographic features rising from the ocean floor. Our results reveal a novel migratory pattern in which whales interrupted their migratory behaviour near oceanic features such as seamounts, which probably provide a suitable environment for breeding, migrating and perhaps feeding humpback whales from New Caledonia. While similar patterns have yet to be documented in other regions, it is likely that seamounts may be used by the species worldwide.

## 2. Material and methods

### 2.1 Satellite tag deployment and biopsy collection

**Figure 1.**
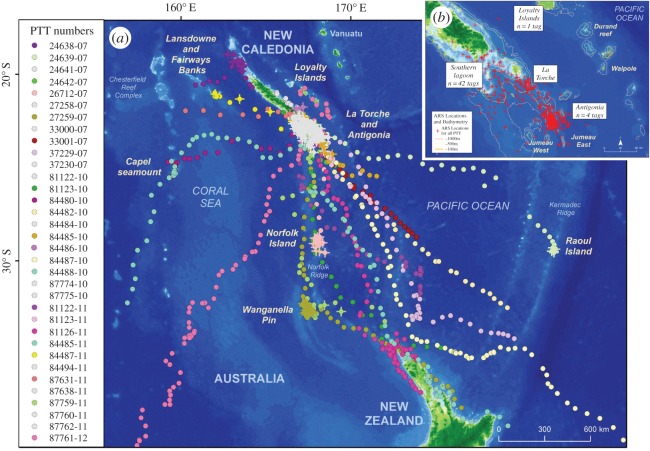
(*a*) Switching state-space model (SSSM)-derived locations for all the tracked whales. Each circle represents a 12 h SSSM location. Area-restricted search (ARS) behaviour locations are indicated by a cross. ARS locations on Antigonia and La Torche have been represented in only one colour. (*b*) Zoomed view of the southern part of New Caledonia with La Torche Knoll as well as Antigonia and others seamounts showing only ARS behaviour locations. ARS behaviour locations are indicated by a red cross for all PTTs grouped.

In order to investigate movements and migration of New Caledonia humpback whales, we deployed 47 Argos satellite-monitored tags in three locations: in the Southern Lagoon (22.5° S–167° E; *n*=42), near Antigonia seamount (23.4° S–168.1° E; *n*=4) and in Lifou, Loyalty Islands (20.9° S–167° E; *n*=1). The tags were deployed during August and September of 2007, 2010, 2011 and 2012 (figure 1). Daily searches for the whales were undertaken using a semi-inflatable boat. We used an 8-m long pole, or a modified pneumatic line thrower (ARTS, Restech) set to pressures ranging from 8 to 12 bars [37,38] to implant the tag into the flank of the whales, in the vicinity of the dorsal fin. The tags corresponded to location-only SPOT5 tube implant transmitters (Molds 177 and 193, Wildlife Computers, Redmond, WA, USA). Tagged whales were identified via photographic documentation of each individual, including right and/or left dorsal fins and underside of the fluke when available. In addition, skin samples for genetic analysis and molecular sex identification [39] were obtained from each whale with a crossbow and custom-made arrows [40].

### 2.2 Argos data processing

Location data were obtained from the ARGOS-CLS system. Each location incorporates a measure of error represented by the following location classes (LC) in descending order of accuracy: 3, 2, 1, 0, A, B and Z [37]. In this study, we removed Argos locations with LC Z and filtered the remaining Argos locations using the statistical package Trip in R [33,41] in order to remove locations that implied unrealistically rapid movements. Removal occurred if travel speed between two consecutive locations exceeded 12 km h^−1^, based on maximum speeds reported for humpback whales [42,43]. Tracks were then reconstructed using these filtered positions.

### 2.3 Switching state-space modelling

A Bayesian switching state-space model (SSSM) [44–46] was applied to the filtered Argos data in order to estimate whale movement parameters and behavioural states from telemetry data. Model fitting was performed using freely available software R (R Development Team, 2011) and WinBugs [47]. The latter uses Markov Chain Monte Carlo (MCMC) methods to compute multi-dimensional integration required in Bayesian statistics to produce a posterior distribution of the parameters of interest. The model was fit to each individual dataset with a total of 40 000 MCMC samples with the first 20 000 discarded as a burn-in. The remaining 20 000 samples were reduced to 2000 by retaining one out of every 10 samples, from which the marginal posterior distribution of parameters of interest was computed.

The SSSM uses a correlation random walk model that switches between two unobservable behavioural states (*b*) thought to represent transiting (*b*=1) and area-restricted search (ARS) (*b*=2). Because *b* is a discrete parameter, the means of the MCMC samples were used to compute behavioural modes for predicted locations following the approach of Jonsen *et al*. [45]. We considered mean estimates below 1.25 and above 1.75 as transiting and ARS behaviours, respectively. These two modes are defined according to travel speed and turning angles. ARS behaviour occurs when a whale reduces its speed of travel and increases turning angle, characteristics which could be indicative of foraging but also of resting or breeding behaviour [46]. We classified mean estimates between 1.25 and 1.75 as uncertain following a conservative approach [45]. Predicted locations and behavioural modes were computed at 12-h intervals.

### 2.4 Environmental data

In order to evaluate potential differences in habitat characteristics between searching and transiting areas, we calculated depth, as well as distance to the 200 m and the 500 m isobaths for each location using the results of SSSM. Depth was obtained from the NOAA ETOPO Bathymetry (https://www.ngdc.noaa.gov/mgg/global/etopo1sources.html). Distance to isobaths was calculated using the Geographic Information System package Arc View 10.0, and its extension Spatial Analyst, from ESRII Corporation. We conducted one-way factorial analysis of variance (ANOVA) on depth and distance to the 200 m and 500 m isobaths to identify the characteristics of each habitat.

### 2.5 Occupancy time

In order to identify areas of higher usage, the SSSM predicted locations were plotted in a grid containing 10×10 km cells that encompassed the area visited by the tagged New Caledonia humpback whales. The average time (in hours) spent by whales in each grid square was computed by multiplying the total number of positions per grid square by 12 (h) and dividing that by the number of individuals that visited each grid cell.

The minimum time spent in areas of interest (in number of days) was calculated using only consecutive positions where the individual remained associated with a given area.

### 2.6 Speed of the whales

A whale was determined to be migrating when it left the southern part of New Caledonia, crossed the 1000 m isobaths and began travelling over deep water. Speed of the tagged whales was calculated by sex and reproductive categories in the breeding ground before migrating and during migration. Categories included males (M), females with calves at the time of deployment (MC) and females without calves (F). Speed was not estimated when fewer than 10 locations were collected. Swimming speed was tested using a two-factorial ANOVA for an effect on reproductive categories and migration followed by a two-by-two post-hoc Neuman–Keuls test.

## 3. Results

The 47 tags were deployed on adult humpback whales, including 13 females accompanied by a calf, eight non-calving females and 26 males. Two tags never transmitted and 11 transmitted for periods of less than 6 days. The remaining 34 tags transmitted for between 5 and 110 days (mean 26.8±19.8 days). A total of 6780 locations were received of which 5004 were retained after filtering. A total of 13 whales (five females versus eight males) were tracked only in their breeding grounds off New Caledonia, but 21 other individuals (11 females and 10 males) continued to be monitored after they had initiated their southbound migrations (table 1).

**Table 1.** Summary of satellite tracking for humpback whales based on SSSM-derived locations estimated every 12 h (sex determined by molecular identification; social category: S, single; P, pair; 2–3, group of two to three adults; MC, mother and calf; MCE, mother with calf and escort; E, escort of a mother and calf pair; CG, competitive group; CG+MC, competitive group with mother and calf; percentage of transiting, ARS and uncertain behaviours locations, n.e.: not estimated, n.a.: not available).

PTT	date deployment	tag duration (days)	sex	minimum total distance (km)	social category	mean speed (km h^−1^)	breeding ground mean speed (km h^−1^)	migratory mean speed (km h^−1^)	ARS (%)	transiting (%)	uncertain (%)
24638-07	19/08/2007	26	F	1860	MCE	2.9	2.7	n.e.	25.0	25.0	50.0
24639-07	01/09/2007	39	M	2942	CG+MC	3.2	2.2	4.3	54.9	29.6	15.5
24641-07	27/08/2007	13.5	M	769	CG	2.5	2.5	n.a.	74.1	0.0	26.0
24642-07	10/09/2007	18.5	F	938	P	2.2	1.9	n.e.	8.1	5.4	86.5
26712-07	27/08/2007	21.5	M	1533	S	3.0	2.0	3.8	65.1	20.9	14.0
27258-07	02/09/2007	8	M	270	CG	1.5	1.5	n.a.	37.5	0.0	62.5
27259-07	09/09/2007	52.5	F	3340	MC	2.7	2.5	2.7	47.0	46.1	6.9
33000-07	02/09/2007	7.5	M	608	CG	3.7	3.7	n.a.	92.9	0.0	7.1
33001-07	07/09/2007	14.5	M	1132	P	3.6	n.e.	3.6	7.4	0.0	92.6
37229-07	10/09/2007	22	F	2131	P	4.2	n.e.	4.3	0.0	95.3	4.7
37230-07	11/09/2007	43.5	F	2114	MC	2.0	2.0	n.a.	77.4	0.0	22.6
81122-10	07/08/2010	24.5	M	820	P	1.4	1.4	n.a.	87.5	0.0	12.5
81123-10	08/08/2010	36.5	F	2524	MCE	3.1	1.9	4.0	1.5	34.3	64.2
84480-10	15/08/2010	16	F	1379	CG	3.8	n.e.	4.1	0.0	80.0	20.0
84482-10	15/08/2010	19	M	1736	S	3.9	n.e.	4.5	0.0	97.4	2.6
84484-10	15/08/2010	20.5	M	467	MC+MC	1.1	1.1	n.a.	86.5	0.0	13.5
84485-10	27/08/2010	17.5	M	1173	P	2.9	2.5	3.5	70.6	0.0	29.4
84486-10	15/08/2010	18.5	M	649	CG+MC	1.5	1.5	n.a.	8.6	2.9	88.5
84487-10	16/08/2010	51.5	M	4738	P	3.9	n.e.	4.1	0.0	100	0.0
84488-10	17/08/2010	19	M	2050	P	4.7	n.e.	4.9	0.0	97.3	2.7
87774-10	02/08/2010	35.5	M	1247	2–3	1.5	1.5	n.a.	97.1	0.0	2.9
87775-10	03/08/2010	16.5	F	584	P	1.6	1.6	n.a.	93.8	0.0	6.2
81122-11	17/09/2011	20	F	1733	CG	3.7	n.e.	3.8	0.0	85	15.0
81123-11	29/08/2011	17	F	1092	MC	2.9	1.9	4.2	0.0	90.6	9.4
81126-11	08/09/2011	54.5	F	3397	S	2.6	n.e.	2.6	0.9	64.5	34.6
84485-11	31/08/2011	48	F	2573	MC	2.3	n.e.	2.3	0.0	98.9	1.1
84487-11	25/08/2011	16	M	1097	P	3.0	1.9	4.0	13.3	10	76.7
84494-11	01/09/2011	15.5	F	929	MC	2.9	2.9	n.a.	96.1	0.0	3.9
87631-11	27/08/2011	11.5	M	1163	P	4.4	2.9	n.e.	4.4	39.1	56.5
87638-11	29/08/2011	23.5	F	1001	MC	1.8	1.8	n.a.	28.3	0.0	71.7
87759-11	30/08/2011	21.5	M	2358	P	4.5	n.e.	4.6	5.0	27.5	67.5
87760-11	31/08/2011	31	F	1583	MC	2.2	2.2	n.a.	23.7	5.1	71.2
87762-11	16/09/2011	9	M	264	E	1.3	1.3	n.a.	94.4	0.0	5.6
87761-12	28/09/2012	110	F	8540	MC	3.2	2.3	3.3	4.1	86.7	9.2

The recorded minimum distance travelled by the whales was between 270 and 8540 km with a general mean speed of 2.83±2.11 km h^−1^. In the breeding ground, a mean speed of 2.01±1.63 km h^−1^ was recorded (2.10±1.79, 2.18±1.67 and 1.85±1.55 km h^−1^, respectively, for MC, F and M); by contrast, migratory speeds averaged 3.53±2.22 km h^−1^ (3.33±2.10, 3.09±2.03 and 4.30±2.36 km h^−1^, respectively, for MC, F and M). Speed was significantly different for all social categories during migration compared with on the breeding ground (*p*<0.001; electronic supplementary material, table S1). The speed of migrating males was significantly greater than that of females with or without calves (*p*<0.001), whereas no differences was observed among reproductive categories (*p*>0.005) within the breeding ground (electronic supplementary material, table S2).

The tagged whales moved in a wide range of directions when they left the breeding ground of New Caledonia (figure 1*a*). Approximately three-quarters of them headed to the south or southeast, while the remaining individuals took a different route towards the west, highlighting the importance of the Coral Sea and of the Chesterfield reef complex (figure 1*a*). Neither sex nor social role at the time of tagging influenced choice of direction: both sexes and reproductive classes were found following all of the observed routes; three males and two females (one with a calf) followed the western direction, while seven males and nine females (five with calves) took a south–southeasterly route.

Our tracking data revealed some offshore habitats that appear to be important for humpback whales, with both the SSSM and occupancy time indicating significant use of these areas. In most cases, the two methods showed consistent results; however, as this was not always the case, we decided to present both methods because they complement each other and both provide relevant information.

The SSSM distinguished between transiting (45% of locations) and ARS (30% of locations); behavioural modes for the remaining 26% of locations were classified as uncertain. Environmental parameters defined a shallower environment for ARS locations than for transiting, with a mean depth of 650±758 m versus 2293±994 m (*F*=342, *p*<0.001). They were also located significantly closer to the −200 and −500 m isobaths than locations classified as transit (49±296 km versus 360±669 km and 47 km±295 versus 324±668 km, respectively; *F*=57, *p*<0.001 and *F*=46, *p*<0.001).

Approximately three-quarters of the ARS behavioural locations were situated less than 50 km from specific geographical features. Offshore seamounts and shallow environments (figure 1*a*,*b*) were identified as important areas by the SSSM. Within a radius of 50 km from these features, 63% of the locations estimated by the SSSM corresponded to ARS behaviour while only 9% were classified as transit.

**Figure 2.**
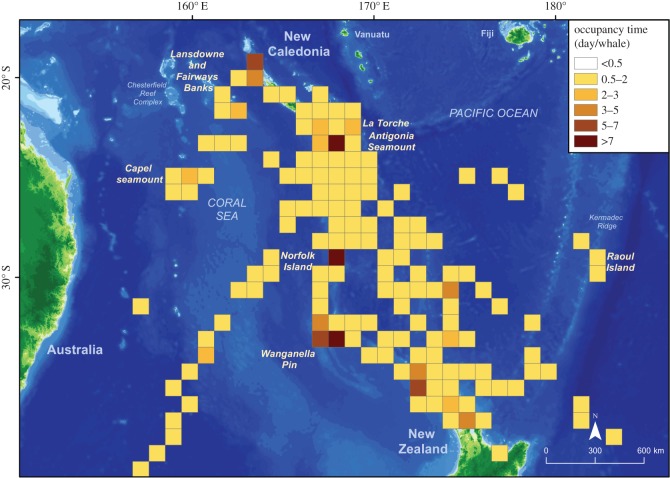
Time of occupancy defined as the total number of hours spent in each 100 km^2^ grid square divided by the number of whales in that grid square, and expressed in days/whale.

SSSM showed that whales engaged in ARS behaviour in association with several features. These included the seamounts of Antigonia and Wanganella Pin, the knoll of La Torche and the vicinity of Norfolk and Raoul islands (figure 1*a*,*b*). However, this method did not identify other features such as the Capel seamount or the northern coastal waters of New Zealand; by contrast, these were highlighted by the occupancy time analysis (figure 2). Finally, this latter method confirms the use by humpback whales of the Northern Lagoon of New Caledonia and the shallow Fairway and Lansdowne Banks, neither of which were clearly identified using SSSM.

That whales are not just passing through but changing their behaviour in the vicinity of these features is suggested by the occurrence of erratic movements, low speed or extended periods of time spent there. A high proportion (68%) of the tagged whales displayed such behavioural changes within the breeding ground of New Caledonia (39%) or on their vernal migration (62%) through middle latitudes (18°–37° S).

Finally, the high percentage of tagged whales (74%) that congregated on offshore seamounts both during the breeding season and during migration suggests that this particular geographical feature is of considerable importance as a habitat for the breeding population of humpback whales of New Caledonia. The occupancy time further highlights the likely importance of these geographical features. The offshore seamounts of Antigonia and Wanganella Pin appear to be areas in which occupancy was prolonged, on average exceeding 7 days/whale (figure 1*b*). The longest stay was found on Antigonia seamount (22.5 days; mean 9.4±5.8 days), followed by Wanganella Pin (17 days). There, the mean speed of the tagged whales was 1.3±1.0 km h^−1^ and 1.5±1.0 km h^−1^ for Antigonia and Wanganella Pin, respectively. A shorter amount of time was spent on Capel seamount, with occupancy being 3–5 days/whale.

## 4. Discussion

### 4.1 Multiple paths and a widely dispersed migratory pattern

The use of multiple paths by the New Caledonian humpback whales leaving their breeding ground contrasts somewhat with what has been described elsewhere. For example, whales migrating from their breeding grounds off the eastern and western coasts of South America and Africa moved directly to their feeding grounds near the Scotia Sea (approx. 55° S) and the Bouvet Islands (approx. 54° S) in a nearly straight line, without noticeable stops and in relatively narrow corridors [30,32,33,37]. Similarly constrained movements have been observed among whales migrating from the breeding grounds of Madagascar heading in the direction of the Crozet Plateau (approx. 46° S) and Prince Edward Islands (approx. 47° S) [31]. In Brazil, paths have been shown to be remarkably consistent among individuals. They use a narrow corridor 600–800 km wide, with some tracks extensively overlapping for part of the migration [33].

By contrast, whales departing from New Caledonia on their southern migration travelled along widely dispersed migratory paths spread longitudinally over 1600 km between the Kermadec Ridge and the Norfolk Ridge, and showed only limited overlap in the latter region. Humpback whales tagged just before leaving the southern coast of Australia also showed dispersed southbound migratory paths [42]. Matches of photographically identified individual humpbacks between the breeding areas of American Samoa and high-latitude feeding grounds of the Antarctic Peninsula also indicate considerable longitudinal displacement [48]. Furthermore, a number of movements reported from the return of Discovery marks^1^ deployed during the commercial whaling, even if limited, suggest that whales from breeding grounds off eastern Australia and Tonga have a broad longitudinal distribution across several Antarctic feeding areas [49]. The wide range of migratory paths used by the humpback whales tagged in New Caledonia during their southern migration is consistent with the connections inferred from this Discovery marking. Collectively, these observations may indicate a broad spread of migration patterns among South Pacific whales in their movements to Antarctic feeding grounds.

In the Cook Islands tagged whales have also been observed spreading out, but this movement does not seem to correspond to a southern migration as all the whales were heading in a westerly or northwesterly direction, with some of them reaching the breeding ground of Samoa [50]. Similarly, it is probable that the whales leaving New Caledonia in the direction of the Coral Sea are not yet on their southern migration but are heading to other low-latitude areas; this highlights the importance of the Coral Sea and of the Chesterfield reef complex which could represent a previously overlooked breeding ground. The Chesterfield reef complex was historically known as a hotspot for American sail-based whalers in the nineteenth century [51,52], and there has been speculation regarding whether it remains as a breeding destination for this species [53]. Recent vessel surveys undertaken in this area suggest that it could be used for reproduction [51], but more data are necessary to evaluate this hypothesis.

Finally, the absence of tagged whales travelling on to other known wintering destinations within Oceania corroborates the comparison of photo-id and genotypes that have documented a low rate of exchange between New Caledonia and other areas [54,55]. The tagging data support the idea that the New Caledonian whales are not connected with other tropical regions within the South Pacific.

The average speed of the New Caledonian migrating whales (3.53±2.22 km h^−1^) is similar to that reported for the Brazilian population (3.34 km h^−1^) [33,37] and is slightly lower than values given for migrating humpbacks in the Northern Hemisphere (4.5, 4.3 and 4.0 km h^−1^) [34,56,57]. The average speeds estimated for the migrating New Caledonian females, with or without calves (respectively, 3.09 and 3.33 km h^−1^), are slightly lower than those reported for whales tagged in the North Atlantic Ocean (3.9 and 4.9 km h^−1^) [56].

### 4.2 Offshore habitats inferred from tracking data

The tracking data highlighted that migrating whales commonly associate with various shallow geographical features such as seamounts, banks or coastal areas; these include Antigonia and Capel seamounts, Wangella Pin and La Torche Knoll, Fairway and Lansdowne Banks, Norfolk and Raoul islands, and the northern shore of New Zealand. That 28 individuals passed through La Torche Knoll and Antigonia seamount (figure 1*b*) suggests that these offshore features are important habitats for humpback whales breeding in New Caledonia. During the southern migration, the areas of interest appeared to be more dispersed than those on the breeding ground, as the whales followed different paths. Several individuals migrated through Wanganella Pin (one male, one female with calf), Capel seamount (one male, one female without calf), Fairway and Lansdowne Banks (one male, one female with calf) or Norfolk Island (one male, one female with calf). By contrast, only one whale passed by Raoul Island (one male) or the shallow bank south of the Loyalty Islands (one female with calf).

### 4.3 Satellite tracking revealed a change in whale behaviour around seamounts

The tracking data indicated that whales not only migrated by seamounts and some other types of oceanic features such as islands and banks, but that they remained in and actively used some of these locations.

A cluster of seamounts occur in southern New Caledonian EEZ waters (e.g. Jumeau East and West, Stylaster), but interestingly only La Torche and Antigonia were intensively used by the tagged whales. Situated in the open ocean, 36 and 110 km (respectively) of the southern island of the archipelago, these two features have a minimum depth of 30 and 60 m. This shallow bathymetry may explain the whales’ presence in these locations, as other neighbouring seamounts are a few hundred metres below the sea surface. With a minimum depth of 82 m under the sea surface, the Wanganella Pin in Basin de La Gazelle is also a shallow seamount, as is Capel seamount, which is slightly deeper at 130 m. Depths of less than 100 m are preferentially used by humpback whales in breeding areas, supporting the hypothesis that an aggregation effect is limited to shallow seamounts as described for other marine species [11].

The amount of time spent in such locations raises another question regarding the potential use of seamounts. The only previous observation of use of a seamount by a migrating humpback whale (the Kermit-Roosevelt seamount in the North Pacific Ocean, 39° N, 146° W) occurred during a period of high oceanographic productivity, suggesting the potential for foraging in such regions [2]. However, this behaviour has not to date been documented in the areas of interest highlighted in our paper, and the significantly higher latitude of Kermit-Roosevelt may substantially influence the productivity of that area. However, seamounts may also have other roles, including as areas for breeding activities; both singing by males and competitive behaviour have been reported on migration routes [58,59]. Seamounts could also act as a navigational cue or landmark on migratory corridors; these features often have distinct geomagnetic signatures, which may be used by species that are known to detect magnetic fields during migration [60–62]. Finally, they could represent resting stops.

## 5. Conclusion

The migratory pattern of the New Caledonian humpback whales differs from those of some other known populations due to congregation on seamounts and other oceanic features, multiple directions upon departure from their breeding grounds and the geographical spread of the migratory ‘corridor’. To date, this type of variable migratory pattern has not been reported in other Southern Hemisphere humpback whale populations.

Seamounts and some other oceanographic features such as islands and banks are extensively used by the humpback whales wintering in New Caledonia, and may play an important role for this endangered population. It is possible that seamounts represent important habitats for other humpback whale populations inhabiting ocean basins where such features are common, as this is the case in the Pacific Ocean where the majority of large seamounts occur. There, the occurrence of seamounts peaks between 30° N and 30° S [63], but studies have been conducted on only a small portion of the potential seamount features and available information is scarce. In the North Pacific, seamounts could potentially be part of a hypothesized unsampled breeding area for the humpback whales that feed in the Aleutians Islands/Bering Sea [64]. By contrast, shallow seamounts are not as common in the South Atlantic, which could explain why whales in this ocean appear more likely to migrate directly to the Antarctic.

The use of seamounts on migratory routes suggests that they play multiple roles as resting areas, navigational landmarks or even supplemental feeding areas for migrating humpback whales. Since a substantial number of seamounts are shallow and in low latitudes, our results suggest that such remote features could represent previously overlooked cryptic habitats for humpback whales. This may have implications for assessment of population structure and for the estimation of abundance, since studies of both could be biased by incomplete coverage of a particular stock’s range. Therefore, further studies are required to assess the extent to which seamounts are used by humpback whales worldwide. Overall, their apparent importance at key stages of this species’ life cycle has significant ecological and management implications, and needs to be taken into account in the design of offshore Marine Protected Areas and other protective measures.

## Supplementary Material

Table S1. Results of the two-factor ANOVA for an effect relating to reproductive categories and migration.

## Supplementary Material

Table S2: Results from Neuman-Keuls Test: probability of Post-Hoc error, mean square = 3.7323, df = 1729.

## Footnotes

1 A Discovery mark was a uniquely numbered stainless steel cylinder fired into a whale from a shotgun. If the whale was subsequently killed by whaling, the mark would often be recovered during processing of the carcass, thus providing two locations (the marking and the recovery) for investigations of movements and population structure.

## Data accessibility

The data supporting this article are available at the Dryad digital repository: http://dx.doi.org/10.5061/dryad.hh205.
