# Supplementary material for: Satellite tracking reveals novel migratory patterns and the importance of seamounts for endangered South Pacific humpback whales
Source: R Soc Open Sci. 2015 Nov 25;2(11):150489. doi: 10.1098/rsos.150489 (PMC4680621; doi:10.1098/rsos.150489)
Supplement: Table S1. Results of the two-factor ANOVA for an effect relating to reproductive categories and migration. [file rsos150489supp1.docx]

SUPPORTING INFORMATION

**Table S1**. Results of the two-factor ANOVA for an effect relating to reproductive categories and migration.

|  | **Sum of square** | **Degree of freedom** | **Mean square** | **F** | **P** |
| --- | --- | --- | --- | --- | --- |
| **Origin** | 10067.87 | 1 | 10067.87 | 2697.48 | < 0.001 |
| **Breeding/Migration** | 751.03 | 1 | 751.03 | 201.22 | < 0.001 |
| **Reproductive category** | 71.72 | 2 | 35.86 | 9.60 | <0.001 |
| **Breeding/Migration*Reproductive categories** | 218.59 | 2 | 109.29 | 29.28 | <0.001 |
| **Error** | 6453.17 | 1729 | 3.73 |  |  |
