# Supplementary material for: Satellite tracking reveals novel migratory patterns and the importance of seamounts for endangered South Pacific humpback whales
Source: R Soc Open Sci. 2015 Nov 25;2(11):150489. doi: 10.1098/rsos.150489 (PMC4680621; doi:10.1098/rsos.150489)
Supplement: Table S2: Results from Neuman-Keuls Test: probability of Post-Hoc error, mean square = 3.7323, df = 1729. [file rsos150489supp2.docx]

**Table S2**: Results from Neuman-Keuls Test: probability of Post-Hoc error, mean square = 3.7323, df = 1729.

| **Breeding/ Migration** | **Reproductive category** | **F/BG** | **M/BG** | **MC/BG** | **F/M** | **M/M** | **MC** |
| --- | --- | --- | --- | --- | --- | --- | --- |
| BG | FC |  | 0.182 | 0.669 | 0.000 | 0.000 | 0.000 |
| BG | M | 0.182 |  | 0.182 | 0.000 | 0.000 | 0.000 |
| BG | F | 0.669 | 0.182 |  | 0.000 | 0.000 | 0.000 |
| M | MC | 0.000 | 0.000 | 0.000 |  | 0.000 | 0.196 |
| M | M | 0.000 | 0.000 | 0.000 | 0.000 |  | 0.000 |
| M | F | 0.000 | 0.000 | 0.000 | 0.196 | 0.000 |  |
